# The Role of MIF in Type 1 and Type 2 Diabetes Mellitus

**DOI:** 10.1155/2014/804519

**Published:** 2014-01-02

**Authors:** Yuriko I. Sánchez-Zamora, Miriam Rodriguez-Sosa

**Affiliations:** Unidad de Biomedicina, Facultad de Estudios Superiores-Iztacala, Universidad Nacional Autónoma de México, Avenida de los Barrios No. 1, Los Reyes Iztacala, 54090 Tlalnepantla, MEX, Mexico

## Abstract

Autoimmunity and chronic low-grade inflammation are hallmarks of diabetes mellitus type one (T1DM) and type two (T2DM), respectively. Both processes are orchestrated by inflammatory cytokines, including the macrophage migration inhibitory factor (MIF). To date, MIF has been implicated in both types of diabetes; therefore, understanding the role of MIF could affect our understanding of the autoimmune or inflammatory responses that influence diabetic pathology. This review highlights our current knowledge about the involvement of MIF in both types of diabetes in the clinical environment and in experimental disease models.

## 1. Introduction

**Figure 1:**
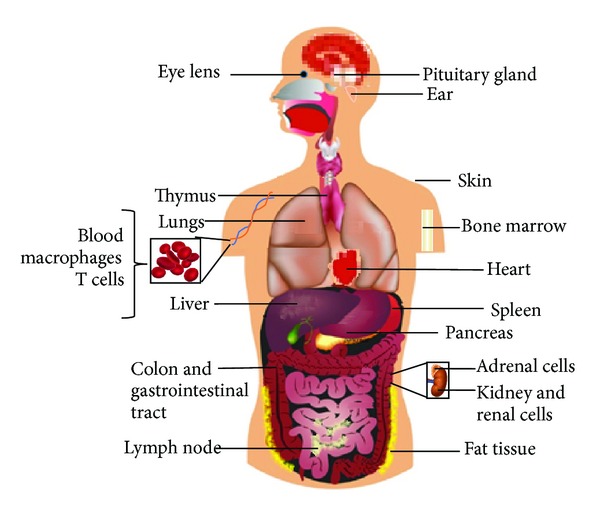
MIF expression pattern. MIF, a cytokine, is distributed throughout almost the entire body. This is because MIF is part of the innate immune system or first line of immune defense.

MIF was originally reported in 1966 by two different groups and was described as a T cell derived cytokine that inhibited the random migration of macrophages *in vitro* and promoted macrophage accumulation during delayed-type hypersensitivity reactions [1, 2]. Human and mouse MIF genes are 90% homologous; MIF protein has a molecular weight of 12.5 kDa [3]. MIF is an evolutionarily conserved molecule that is constitutively expressed in many tissues and cells (Figure 1).

Moreover, MIF is stored in intracellular pools and therefore does not require immediate synthesis before secretion. MIF lacks an aminoterminal leader sequence; this indicates that MIF is released from cells through a nonconventional protein-secretion pathway [3].

After the discovery of MIF, several studies were conducted to establish its role in the immune response [4–6]. However, not until 1990 was MIF recognized as the first molecule to arrive at the inflammation site and the factor that likely determines the degree of cellular inflammation [7]. Different experimental strategies, including anti-MIF antibodies and knockout (KO) and transgenic MIF mice (MIF-Tg), have been used to establish that MIF counterregulates the immunosuppressive effects of steroids and to implicate MIF in tumor necrosis factor (TNF*α*) and nitric oxide (NO) production [8]; additionally, MIF was found to possess growth factor activity [9], overregulate the expression of Toll-like receptor (TLR)-4 on antigen-presenting cells [10], sustain macrophage proinflammatory abilities by inhibiting p53, and also possess tautomerase and oxidoreductase activities [11].

All the above-described inherent properties permitted the recognition of MIF as a critical molecule in proinflammatory innate immune responses and the restriction of certain parasite infections [12–14]. Additionally, MIF involvement has been demonstrated in immunological and inflammatory diseases [15, 16] such as septic shock [17], cancer [18], and chronic diseases including bowel disease [19], rheumatoid arthritis [20–22], colitis [23], obesity [24–26], and diabetes [25, 27, 28]. More recently, MIF was proposed as a diagnostic biomarker for autoimmune diseases such as arthritis, ulcerative colitis, and diabetes [23, 29, 30].

In this review, we will focus on some of the properties that have been conferred upon MIF with regard to the development and maintenance of T1DM and T2DM. We will discuss the data that have been collected in clinical studies and studies of MIF-KO mice and other protocols in which MIF has been proposed as a therapeutic diabetes mellitus pathological target.

## 2. MIF and Diabetes

### 2.1. Diabetes

This disease comprises a group of metabolic diseases that are characterized by hyperglycemia, which is associated with damage to and/or malfunction or failure of various organs, including the eyes, kidneys, nerves, heart, and blood vessels, among others. The causes of this disease range from autoimmune or metabolic abnormalities to deficiencies in insulin activity and secretion [31]. Currently, there is no cure for this chronic degenerative disease; however, the constant development of knowledge helps us to better understand the disease etiology and potential therapeutical targets that, when combined, could lead to good symptom and disease control [31].

### 2.2. MIF and T1DM or Insulin-Dependent Diabetes Mellitus (IDDM)

According to the American Diabetes Association, T1DM patients comprise only 5–10% of those with diabetes [26]. This disease is a multifactorial, organ-specific autoimmune disease that occurs in genetically susceptible individuals [32]. T1DM is the result of the autoimmune destruction of pancreatic islet *β* cells by infiltrating immune cells (insulitis); this occurs due to a failure in immune tolerance because the organism has had contact with specific viruses [33] such as cytomegalovirus [34] or with food molecules that caused molecular mimicry [35]. The common autoantigens recognized in this disease are insulin, glutamate decarboxylase 65 (GAD65), and the islet antigens IA-2 and IA-2*β* [36, 37]. During insulitis, high levels of proinflammatory cytokines, including IL-1*β*, TNF*α*, IL-12, MIF, and IFN*γ*, are secreted by effector T cells to trigger the *β* cell destruction process [32]. MIF is considered one of the most common factors in autoimmunity [38]. In humans with T1DM, blood MIF concentrations were found to be high, compared to those in healthy controls [39]; normal plasma MIF concentrations in healthy humans range from 2.3 to 8.4 ng/mL [40]. In contrast, plasma MIF concentrations dramatically change from 5 ng/mL to 1 ng/mL after islet transplantation [41]. Also high MIF concentrations are associated with a subsequent loss of islet graft function [41]. IL-1*β* and TNF-*α* are expressed at high levels along with advanced type one diabetes complications such as ketoacidosis [42], and thus it is possible that high levels of MIF are also expressed at this point in the disease. MIF studies were facilitated by the development of MIF-KO mice in 1999 [43]. Using these mice as an efficient tool, MIF was shown to be an important molecule in early syngeneic islet transplantation function, and blocking of MIF resulted in transplant success [44]. Additionally, we know that MIF participates in T1DM by controlling the functional activities of monocytes/macrophages and T cells and modulating their abilities to secrete proinflammatory molecules [45]. Furthermore, MIF has been recognized as important molecule to the development of T1DM complications such as cardiac dysfunction, which is associated with AMPK signaling [46], and diabetic foot disease [47] and is known to promote inflammatory cytokine and palmitic acid-induced pancreatic islet apoptosis [48, 49]. After successful antibody and pharmacological inhibitor-mediated MIF neutralization, MIF was proposed as a new target strategy for the treatment of T1DM [45, 50].

**Figure 2:**
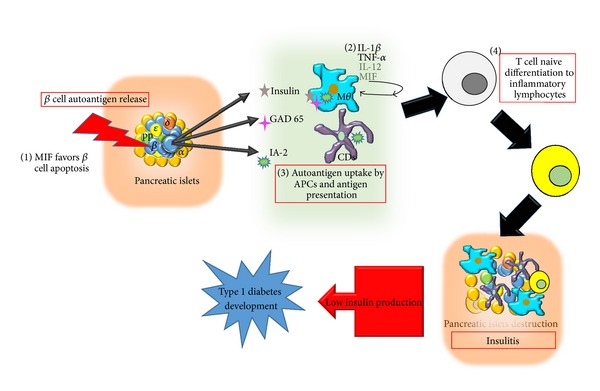
MIF involvement in T1DM development. (1) MIF promotes pancreatic *β* cell apoptosis. (2) MIF promotes the production of inflammatory cytokines such as IL-1*β*, TNF-*α*, and IL-12. (3) MIF favors autoantigen presentation. (4) MIF promotes the activation of an inflammatory response, leading to insulitis.

The involvement of MIF in T1DM is summarized in Figure 2. With the above-outlined information, we can conclude that the participation of MIF in the pathology of T1DM is a well-documented fact; however, we do not know the exact point in disease development at which MIF exerts the most influence. Considering that the insulitis process marks the beginning of the disease and is an autoimmune inflammatory process, we propose the hypothesis that MIF plays an important role in insulitis onset or development. This hypothesis is supported by studies in which MIF was found to play important roles in the processes of antigen presentation and inflammatory cell activation [13, 51]. However, additional studies should be performed to establish the mechanism related to the role of MIF in T1DM.

### 2.3. MIF and T2DM or Noninsulin-Dependent Diabetes Mellitus (NIDDM)

T2DM patients account for 90–95% of all diabetic patients, and this disease is characterized by the presence of insulin resistance and, usually, relative insulin deficiency. The most common risk factors for this type of diabetes are genetic conditions, obesity, lifestyle, and eating habits. Therefore involvement of the inflammatory response is equally important in disease development, hence the reason why the role of MIF has been studied most in T2DM [31].

**Figure 3:**
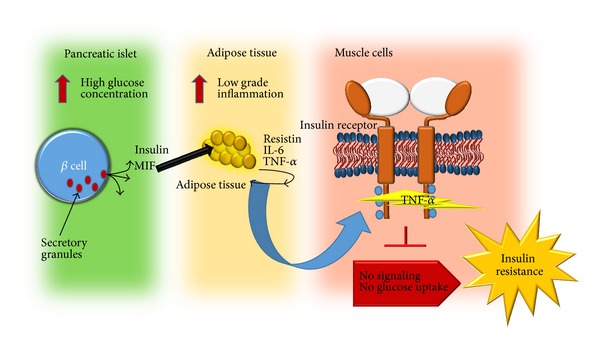
MIF plays an indirect role in T2DM development by promoting the production of proinflammatory cytokines and adipocytokines that are involved in insulin receptor signaling, leading to insulin resistance.

Several clinical studies have shown that the serum MIF levels are elevated in patients with T2DM [52]. In 2006, high blood MIF levels were suggested to precede the onset of T2DM [40]. More recently, both patients with T2DM and those with impaired glucose tolerance were shown to have significantly elevated MIF serum levels [25, 27, 53]. Some data report normal plasma MIF concentrations in healthy humans to range from 2.3 to 8.4 ng/mL [35]. In contrast, plasma MIF concentrations from T2DM subjects range from 7.3 to 15.8 ng/mL. As in T1DM, MIF is highly expressed in T2DM complications such as myocardial damage [54], coronary artery disease [53], diabetic retinopathy [55], obesity [56], and metabolic syndrome [57]. The role of MIF in T2DM has been studied in murine models. Using db/db mice, MIF was suggested as a factor that could initiate the onset of microalbuminuria in diabetic nephropathy [58]. MIF plays an important role in the chronic, obesity-associated adipose tissue inflammation that leads to the development of insulin resistance in MIF-KO mice [59]. Additionally, we previously demonstrated in a NIDDM mice model that MIF could be a therapeutic target for disease treatment. Indeed, two candidate drugs (synthetic inhibitors for MIF) for diabetes treatment were described as very effective in the control of the systemic inflammation and the control of some diabetes symptoms [60]. Additionally, we showed that MIF is important to the production of some adipocytokines such as resistin, which is the most important adipocytokine in the development of insulin resistance [60]. In this type of diabetes, MIF has been suggested to contribute to the disease in an indirect form due to its ability to stimulate the production of other inflammatory cytokines that directly cause damage to muscle cells. This is true for TNF-*α*, which acts on the insulin receptor and prevents the dephosphorylation of insulin receptor substrates, thus blocking receptor signaling and preventing glucose entry into the cell (insulin resistance). Furthermore, MIF also stimulates the production of certain inflammatory adipocytokines, such as resistin and IL-6, which are key molecules in the development of insulin resistance. As demonstrated, MIF influences T2DM development at different levels. In the pancreas, adipose tissue, and muscle cells, the pleiotropic characteristics of MIF are reflected in the different routes that lead to insulin resistance (Figure 3).

## 3. MIF and Pancreatic Beta Cells (*****β***** Cells)

MIF was shown to colocalize in secretory insulin granules within *β* cells and to be released during both phases of insulin secretion. Most importantly, in this regard, MIF appears to have an autocrine, glucose-dependent regulatory effect on insulin secretion [45].

While MIF is related to insulin secretion under homeostatic conditions, altered homeostasis in an organism (such as the presence of inflammation) apparently induces MIF to act differently and become a destructive molecule that can lead to *β* cell apoptosis [48, 61]. Apparently, MIF is not a foreign molecule in the pancreatic *β* cell microenvironment and can act in response to the concentration of glucose and the presence of inflammation.

## 4. Conclusions

Previously, different models were used to show that MIF is a pleiotropic molecule [62–64], and this property is very evident in pancreatic islets. Due to the large number of studies that support this idea, we can conclude that MIF is a proinflammatory cytokine with great importance not only during the course of diabetes but also before the establishment of diabetes and in the risk factors of disease such as obesity. We support the statement that MIF is a therapeutic target and propose that it is necessary to design synthetic MIF inhibitors that could interact with the existing therapies used to treat diabetes.
